# The Effect of Home Visit Program Based on the Continued Kangaroo Mother Care on Maternal Resiliency and Development of Premature Infant: A Randomized Clinical Trial

**DOI:** 10.30476/ijcbnm.2020.86141.1321

**Published:** 2021-01

**Authors:** Marzieh Ghazi, Masoud Zare, Monir Ramezani, Mohammad Heidarzadeh, Hamidreza Behnam Vashani

**Affiliations:** 1 Department of Community Health Nursing, School of Nursing and Midwifery, Mashhad University of Medical Sciences, Mashhad, Iran; 2 Nursing and Midwifery Care Research Center, School of Nursing and Midwifery, Mashhad University of Medical Sciences, Mashhad, Iran; 3 Department of Pediatric, School of Medicine, Tabriz University of Medical Sciences, Tabriz, Iran

**Keywords:** Development, Home visit, Kangaroo mother care, Premature infant, Resilience

## Abstract

**Background:**

Premature birth is a crisis for mothers and affects resilience. Premature babies are at risk for developmental disorders. The Kangaroo Mother Care (KMC) can reduce maternal stress and improve the growth of the baby. This study aimed at assessing the effect of home visit based on the continued KMC on maternal resiliency and development of premature infant.

**Methods:**

This randomized controlled trial conducted on 50 pairs of mothers and premature babies with gestational age of 26-32 weeks who were admitted to Neonatal Intensive Care Unit of Om-al-Banin Hospital, Mashhad, Iran in 2019. The KMC is practiced routinely for all eligible newborns in this hospital. The experimental group continued the KMC one month after discharge at home and received two home visits. Resiliency of the mothers was assessed in admission, discharge, and one month after discharge with the Connor and Davison questionnaire and the development of the newborns was assessed in two months of adjusted age with Ages and Stages Questionnaire (ASQ). Data analysis was performed using SPSS software version 16 and t-test, Mann-Whitney, ANOVA, Friedman, Chi-square, Fishers exact. The significance level was set at P<0.05.

**Results:**

The resiliency score of the mothers one month after discharge was112.50±5.50 and 76.40±5.60 in the experimental and control groups, which was significantly different (P<0.001). The ASQ development score of the newborns in two months of adjusted age was also significantly higher in the experimental than the control group (280.40±15.60vs223.80±22.00) (P<0.001).

**Conclusion:**

The results showed that the home visit program based on the continued KMC was effective in increasing maternal resilience and the development of premature infants. Trial Registration Number IRCT20181121041718N1.

## INTRODUCTION

Every year, approximately 15 million babies are born preterm with a gestational age of less than 37 weeks, and more than 60% of these preterm births occur in Africa and South Asia. ^1^ Prevalence of the preterm birth in Iran is relatively high. It is 9.2% according to a systematic review and meta-analysis. ^2^ Prematurity is also the leading cause of neonatal mortality in Iran. ^3^ According to the Ministry of Health and Medical Education, Iran, 1500 thousand babies are born each year; of them 120,000 are preterm and about 5 to 8 percent of all babies born in Iran are hospitalized right after birth. ^4^ These preterm babies need special care and put a burden on health and social system of the country. ^5^ On the other hand, although advancements in knowledge and technology had helped reduce neonatal mortality, this reduction did not reduce the consequences of low birth weight and prematurity; thus, the need of these newborns has increased. ^6^

Prematurity causes the newborns to be hospitalized right after birth which creates stress in the family; ^6 , 7^ the longer the hospital stay, the more the stress of the parents. Therefore, it is suggested that the hospital stay of the infants should be shortened as much as possible and the required care should be provided at home by the mothers. ^8^

Findings of studies show that home visits help reduce mortality and morbidity of the newborns and improve the feeling of the families. ^9 , 10^ Home visits have positive outcomes for the mother and newborn, increase the mother’s satisfaction, save the costs, and is known as a preferred strategy for health and development of the babies. ^11^

Preterm birth is a crisis for the mothers, makes them feel incompetent, weakens their self-esteem, and lowers their resilience. ^12^ Resiliency is the ability to adapt with disease, pain and important stressful events in the life. ^13^ It includes keeping peace under pressure, having flexibility in facing obstacles, avoiding attrition strategies, and keeping optimism and positive feelings in hardness. ^14^ Most mothers of preterm babies experience a stressful period. Thus, their emotional status and resilience are important. ^15^

The kangaroo mother care (KMC) is a care strategy for preterm babies that was used for the first time in a hospital in Bogota, Colombia in 1978. ^15 , 16^ The KMC provides skin-to-skin contact and breast feeding and also helps cohesion, attachment, mother-infant interactions, and establishment of a coordinated interactive pattern. ^16 , 17^ It increases the capability and self-esteem of the mothers in caring their babies and brings peace to them, returning an increase in their adaptability. ^18^ Since the incubators often unnecessarily separate the preterm babies from their mother, the KMC can be a good alternative for the incubator among those newborns that have overcome the primary problems. ^19^ The available evidence shows that the impacts of KMC include deceased mortality, illness and complications, increased breast feeding, and improvement in growth and physical parameters. It also makes the mothers of preterm babies feel less mental pressure, stress, and guilt feeling. They feel better because they had done more for their baby, and also the better emotional relationship of the mother and baby prevents many of the mood and behavior problems in the future of the baby. ^16 , 19^

Embryonic and extra uterine life together determine the path along which the growth and development of the infant is influenced by genetic, environmental and social factors. ^20^ Premature infants are at risk for a variety of developmental and cognitive disorders, such as motor, cognitive, memory, attention and behavioral problems. ^21^ A premature baby is born during the crucial period of brain development in the third trimester or even earlier. At this time, the brain is rapidly growing, and deprivation of the intrauterine environment and exposure to environmental stresses, such as light, sound, and movement, can affect the brain development. ^22^ How the brain ultimately develops in the intensive care unit (NICU) is largely unclear. The birth of a premature infant and the change of uterine environment to NICU before brain development leads to long-term physical and psychological problems and disability in this high-risk group. ^21^ The method of care in NICU can support and optimize the long-term outcomes of premature neural development in many ways. Parent-child interaction is the cornerstone of premature infant support and behavioral neurodevelopment during both infant and childhood care. Skin-to-skin care, in addition to physiological stabilization and growth, leads to better behavioral neuropathy. These infants have less stressful behaviors and better reflexes. ^23^ In chronic conditions and disease, Transfer from the hospital to home is a critical point in continuity of care and requires good education of the family before discharge. ^8^ Family education about continuing the KMC can reduce the risk of re-admission and the stress of the mothers of preterm newborns. However, the routine procedures of care in Iran neglect the KMC at home. ^16^

Previous studies have reported positive effects of KMC in the NICUs, ^24 , 25^ but, to the best of our knowledge, no published study is available to investigate the impact of KMC and its home visit on resilience of the mothers and development of premature newborns. This study aimed at assessing the effect of home visit based on the continued KMC on maternal resiliency and development of premature infants.

## METHODS

This was a parallel randomized controlled trial on premature newborns and their mothers. Study samples consisted of 50 pairs of mothers and newborns admitted to NICU of the Om-al-Banin hospital, Mashhad, Iran, between March 2019 to November 2019. Newborn babies who need intensive medical care are often put in a special area of the hospital called NICU. 

The sample size was estimated by the mean comparison method and the standard deviation of the two communities. For the variables of maternal resilience from the findings of Salimi’s research ^26^ and variable infant development were the findings of a pilot study. In calculating the minimum sample size, 95% confidence level and 80% test power were considered.

Sample calculation formula:

N=[ (Z_(1-α/2)_+Z_(1-β)_)^2^ × (s_1_^2^+s_2_^2^)]/ (m_1_-m_2_)^2^

Resilience: (S1=8.47, S2=12.62, m1=72.86, m2=56.00, N=7)

Infant development : (S1=15.4, S2=21.7, m1=261.2, m2=238.9, N=12)

Inclusion criteria for the newborns were birth weight of above 1000 g, gestational age of 26 to 32 weeks, stability of clinical conditions of the newborn, lack of congenital anomaly, lack of intra-ventricular hemorrhage grades 3 and 4, and lack of advanced resuscitation history for the baby. Exclusion criteria were instable respiration, need for invasive mechanical ventilation, and newborn death. Inclusion criteria for the mothers were willingness to participate, lack of diagnosed psychiatric disorders, residence in Mashhad city or its suburbs, and lack of self-reported serious tension with husband. Exclusion criteria for the mothers were twin or multiple birth, existence of a chronic disease, stressful event during the study course such as loss of family members or severe illness of another child. 

In this study, 50 mothers and their babies were enrolled using convenience sampling method. Given that the research was done at home, there was no possibility of information leakage between the two groups. For this reason, the allocation of research units to two intervention and control groups was randomly classified as block chain. First, six possible states were created for the four blocks (AABB - ABAB - ABBA - BBAA - BABA - BAAB), and then a draw was made once to arrange the blocks. In addition, A was a sign of the intervention group and B was a sign of the control group. There were a control group and an experimental group each, of which consisting of 25 babies and 25 mothers. There was no attrition in our samples.

**Figure 1:**
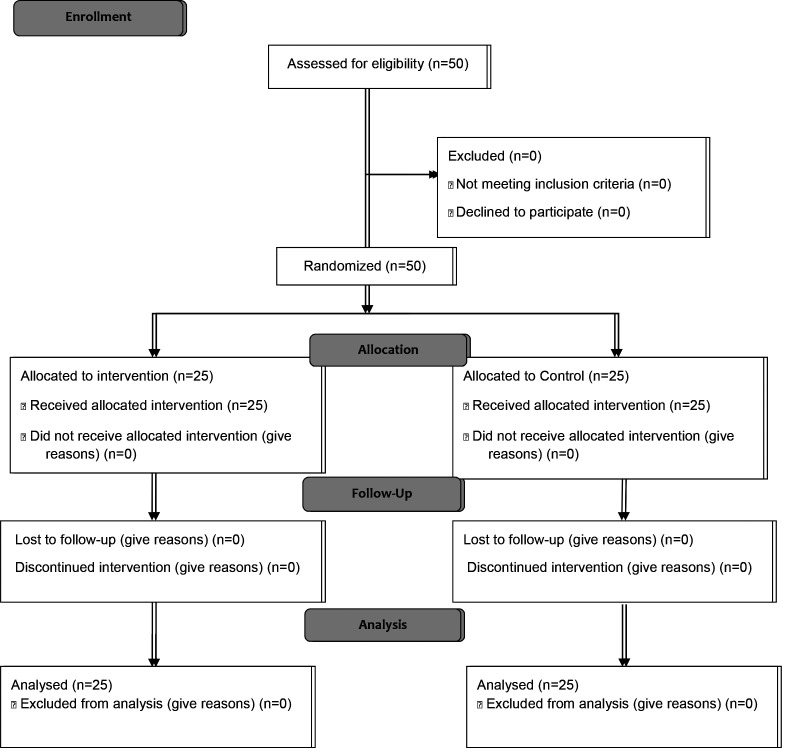
CONSORT Flow Diagram of participants

Finally, the research data were analyzed on 50 people (25 people in each group) (Figure 1).

Study instruments included a researcher-made form for demographic data of the mothers and newborns, the Connor-Davidson Resilience Scale for assessing the resilience of the mothers, and the Ages and Stages Questionnaires (ASQ) version II for assessing the development of the newborns.

Demographic questionnaire included demographic and family characteristics of the research units participating in the study, which includes age, education, parental occupation, family income, insurance, infant sex, gestational age (weeks), birth weight, infant nutrition, type of delivery, number of deliveries, number of abortions, number of pregnancies, number of children, cause of preterm delivery, and wanted or unwanted pregnancy. It was completed based on the information in the file and the interview with the mother.

Connor and Davidson Resilience Scale (CD-RIS): This questionnaire was developed in 2003 by Connor and Davidson after reviewing the research resources of the field of resilience in 1979-1991. Psychometric properties of this scale were performed in six groups of the general population, those referred to the primary care ward, psychiatric outpatients, patients with generalized anxiety disorder, and two groups of patients with post-traumatic stress disorder. This tool has 25 items, each having five options ranging from 1 (completely incorrect) to 5 (always correct). It also has 5 dimensions: 1. Personal competence, high standards, and tenacity; 2. Trust in one’s instincts, tolerance of negative affect, and strengthening effects of stress; 3. Positive acceptance of change and secure relationships; 4. control; and 5. Spiritual influences. The reliability, validity, and factor analytic structure of the scale were evaluated, and reference scores for the study samples were calculated. Reliability was calculated by Cronbach’s alpha method and the reliability coefficient was 0.89; also, test–retest reliability was assessed and demonstrated a high level of agreement, with a correlation coefficient of 0.87. Convergent validity was assessed by correlating the CD-RISC with measures of hardiness, perceived stress, and stress vulnerability, as well as measures of disability and social support. Divergent validity was assessed by correlating the CD-RISC scores with the Arizona Sexual Experience Scale. ^27^ The Persian version was translated in 2005 by Mohammadi et al. Reliability was calculated by Cronbach’s alpha method, with a reliability coefficient of 0.93. The validity and reliability of the Persian resilience form has been examined and confirmed in the preliminary studies of normal and patient samples. ^28^ In the present study, to confirm the validity of this tool, it was given to 7 professors of Mashhad University of Medical Sciences to confirm its compliance with the objectives of the present study. Given that this tool has class options, assessing its reliability in the present study was performed by internal consistency method; the resilience of 15 mothers was measured with this instrument, and then Cronbach’s alpha coefficient was calculated for each of its dimensions. Cronbach’s alpha coefficients were estimated for the overall dimension: Personal competence, high standards, and tenacity (0.88), Trust in one’s instincts, tolerance of negative affect, and strengthening effects of stress (0.86), Positive acceptance of change and secure relationships (0.79), control (0.77), spiritual influences (0.74), and for the total resilience score (0.79).

The ASQ is a parent-completed questionnaire that may be used as a general developmental screen. The ASQ was designed and developed by J. Squires and D. Bricker in 1979 -1980 at the University of Oregon in response to a growing need for early and accurate identification of children who have developmental delays or disorders. The ASQ is a parent reported initial level developmental screening instrument consisting of 20 intervals, each with 30 items in five areas: (1) personal social, (2) gross motor, (3) fine motor, (4) problem solving, and (5) communication for children from 2-60 months. Scoring questions is such that the answer is yes with a score of 10, sometimes a score of 5 and still not a score of zero, and the overall score of the evolution is obtained from the sum of the scores in each field. The ASQ has been translated into several languages, such as Spanish, French, Dutch, Chinese, Norwegian, Hindi, Persian, and Turkish. ^29 , 30^ It has excellent psychometric properties, test-retest reliability of 92%, sensitivity of 87.4%, and specificity of 95.7%. Validity has been examined across different cultures and communities across the world. ASQ is an easy test that can be completed by parents in 12-18 minutes. ^29 , 31^ The Persian version was translated by Sajedi et al. in 2006 and its reliability, determined by Cronbach’s alpha, ranged from 0.76 to 0.86 and the inter-rater reliability was 0.93. The validity determined by factor analysis was satisfactory. ^32^ In the present study, to confirm the validity of this tool, we sent it to 7 professors of Mashhad University of Medical Sciences to confirm its compliance with the objectives of the present study. To determine the reliability of this tool, the internal consistency method was used in such a way that 15 infants were evaluated at the same time in terms of different dimensions of ASQ instrument and then Cronbach’s alpha coefficient was estimated. Cronbach’s alpha coefficients for communication dimensions were 93%, large movements 88%, fine movements, 86%, problem solving 77%, social individual 84%, and overall score of ASQ was 82%.

After obtaining informed consent from the mothers and explaining the goals of the research for them, the resilience scale was filled by the mothers in both groups after admission to the NICU. After removing advanced non-invasive mechanical ventilation equipment from the babies and being eligible for the KMC, all babies in both control and experimental groups received KMC as a routine care in the study NICU. The routine KMC in this NICU is at least three times a day, lasting for 30 to 40 minutes. The KMC is practiced as described here: the mother wears special cloth for the KMC, the baby becomes naked and put on his/her mother’s chest between the breasts in an upright position, the head and chest of the baby is placed on the mother’s chest, abdominal surface of the baby is in contact with the upper abdominal surface of the mother, the head of the baby is turned to one side to help him/her breath and have eye-contact with the mother, and the legs and arms are folded in a frog position. 

The resilience scale was filled again by the mothers in both groups at the time of discharge from the NICU. The experimental group continued the KMC after discharge, at home, for one month and at least three times a day, but the control group did not. A checklist was used to observe the care provided at home by the mothers for the babies in both groups. The checklist included 30 rows for 30 days and three columns for three times of possible KMC each day. The mothers were trained to put a check mark at the table for each time of KMC. In the control group, since the mothers did not continue KMC after discharge, they did not record a case in the checklist. The experimental group received two home visits that lasted for at least 30 minutes in the 15th and 30th days after discharge from hospital. The home visits were conducted by an experienced NICU nurse (One of the researchers). In these visits, the care performance of the mothers was examined by the nurse researcher including these items: assessing the feeding of the baby with an emphasis on exclusive breast feeding, assessing home environment to be safe and proper for care of the premature newborn and the KMC, optimizing the health and development of the newborn, and assessing the interactions of the family with health care providers. 

In the first two months after discharge, the mothers in both groups were phone called every 15 days to know about the status of the newborn and the exclusive breast feeding. The ASQ was filled out by the mothers in both groups at the adjusted age of two months after the baby was 40 weeks. The Statistical Package for Social Sciences (SPSS) version 16 was used for statistical analysis. Independent Samples t-test for variables with normal distribution, Mann Whitney for variables with abnormal distribution, and Chi square tests for qualitative variables were applied to assess the possible differences. The Friedman test is a nonparametric test used to compare the average ranking of different groups, and ANOVA with repeated measure was used to sequentially measure a specific variable in each observation and several different time positions. All statistical tests were performed at a significance level of 0.05. Blinding in this study was only done for statistical analysis.

The study protocol was reviewed and approved by the Ethics Committee of Mashhad University of Medical Sciences with the code of IR.MUMS.NURSE.REC.1397.074. Informed consent of all participating mothers was obtained. 

## RESULTS

Mean gestational age and mean birth weight of the newborns in the experimental and control groups were not significantly different (P=0.17), (P=0.06). In terms of gender, the babies in both groups were not significantly different (P=1.00). All infants participating in this study were exclusively breastfed.

To examine the normal distribution of quantitative demographic variables, we used Kolmogorov-Smirnov and Shapiro-Wilk tests and a significance level of 5% was considered. The results showed that quantitative demographic variables including maternal age, paternal age and birth weight had a normal distribution and gestational age did not have a normal distribution.

Table 1 compares demographic variables in the experimental and control groups; the test results showed that the two experimental and control groups were not significantly different and were homogeneous.

**Table 1:** Comparison of demographic variables in the experimental and control groups

Variable		Experimental group N (%)	Control group N (%)	P value
Sex of infant	Male	15 (60)	15 (60)	*P=1.00
	Female	10 (40)	10 (40)	
Covered by insurance	Yes	24 (96)	25 (100)	**P=1.00
	No	1 (4)	0 (0)	
Pregnancy acceptance	Wanted	23 (92)	21 (84)	**P=0.66
	Unwanted	2 (8)	4 (16)	
Type of delivery	Vaginal delivery	13 (52)	15 (60)	*P=0.56
	Caesarean section	12 (48)	10 (40)	
Number of deliveries	One	18 (72)	14 (56)	*P=0.12
	Two	6 (24)	5 (20)	
	Three or more	1 (4)	6 (24)	
Gravida	One	10 (40)	10 (40)	*P=0.52
	Two	7 (28)	4 (16)	
	Three or more	8 (32)	11 (44)	
Number of abortions	None	11 (44)	15 (60)	*P=0.32
	One	11 (44)	6 (24)	
	Two or more	3 (12)	4 (16)	
Number of previous children	One	18 (72)	15 (60)	*P=0.22
	Two	6 (24)	5 (20)	
	Three or more	1 (4)	5 (20)	
Mother’s job	Employee	2 (8)	3 (12)	*P=1.00
	Manual worker	0 (0)	1 (4)	
	Student	1 (4)	0 (0)	
	Housewife	22 (88)	21 (84)	
Father’s job	Employee	4 (16)	3 (12)	*P=0.72
	Freelance jobs	15 (60)	13 (52)	
	Manual worker	6 (24)	9 (36)	
Cause of preterm birth	Maternal hypertension	5 (20)	0 (0)	*P=0.35
	Premature rupture of membranes	10 (40)	11 (44)	
	Infectious disease of the mother	1 (4)	1 (4)	
	Gestational Diabetes	1 (4)	1 (4)	
	Fetal or placental problems	2 (8)	3 (12)	
	The onset of labor contractions	6 (24)	9 (36)	
Mother’s education	High school	3 (12)	7 (28)	*P=0.31
	Diploma	13 (52)	12 (48)	
	University	9 (36)	6 (24)	
Father’s education	High school	5 (20)	12 (48)	*P=0.08
	Diploma	13 (52)	10 (40)	
	University	7 (28)	3 (12)	
		Mean ±SD	Mean ±SD	
Gestational age (week)		29.70 ±1.20	30.10 ±1.10	***P=0.17
Birth weight (g)		1222.40±187.70	1328.00±200.50	****P=0.06
Age of mother (years)		28.00±4.70	26.90±4.70	****P=0.41
Age of father (years)		32.90±5.00	30.40±5.10	****P=0.08

* Chi square test;

** Fishers exact test;

*** Mann Whitney test;

**** Independent t-test

The mean±SD resilience total score of the mothers at the admission day was 75.80±3.40 for the experimental group and 74.30±2.20 for the control group. Independent Samples t-test showed no difference between the two groups’ resilience total scores at the baseline (P=0.07). The resilience total scores on the discharge day were 94.00±3.80 for the experimental group and 92.80±3.10 for the control group, which was not significantly different (P=0.21). The total scores at one month after discharge were 112.50±5.50 for the experimental group and 76.40±5.60for the control group, which were significantly different according to Independent Samples t-test (P<0.001). 

The intra-group comparisons using the repeated measures analysis of variance (Repeated Measured ANOVA) found that the resilience total scores in the experimental group had increased significantly (P<0.001), but they were not significantly different in the control group (p=0.11). The Bonferroni post-hoc test showed that in the experimental group the differences between the total scores of the admission and discharge (P=0.001), between total scores of the admission and one month after discharge (P=0.001), and between total scores of the discharge and one month after discharge (P=0.001) were statistically significant.

The Resilience dimension scores were also compared. The Independent Samples t-test and the Mann Whitney test were applied for this comparison between the experimental and the control groups. As Table 2 shows, the scores were significantly different in all five dimensions. 

**Table 2:** Comparisons of scores of the mothers’ resilience in the experimental and control groups in the five dimensions of the Connor -Davidson Resilience Scale

Dimension of the resilience scale	Stage of the study	Mean±SD		P value
		Experimental group	Control group	
Personal competence, high standards, and tenacity	Admission	22.80±2.00	22.40±2.10	*P=0.49
	Discharge day	26.30±1.40	25.60±1.40	*P=0.09
	One month later	35.40±2.90	23.40±2.30	*P<0.001
P value		***P<0.001	***P<0.001	
Trust in one’s instincts, tolerance of negative affect, and strengthening effects of stress	Admission	19.00±2.60	18.10±2.40	**P=0.11
	Discharge day	24.80±1.60	24.00±1.50	*P=0.06
	One month later	30.40±2.80	17.60±2.30	**P<0.001
P value		****P<0.001	****P<0.001	
Positive acceptance of change and secure relationships	Admission	18.00±1.80	17.20±1.10	**P=0.17
	Discharge day	20.80±1.80	20.10±1.70	**P=0.15
	One month later	23.20±1.30	17.50±2.20	**P<0.001
P value		****P<0.001	****P<0.001	
Control	Admission	9.60±1.00	9.00±1.10	**P=0.10
	Discharge day	13.00±1.10	12.90±1.00	**P=0.54
	One month later	13.90±0.90	8.90±1.00	**P<0.001
P value		****P<0.001	****P<0.001	
Spiritual influences	Admission	6.40±1.30	6.20±1.30	*P=0.60
	Discharge day	8.60±0.90	8.10±1.00	*P=0.08
	One month later	9.60±0.50	7.20±1.50	**P<0.001
P value		***P<0.001	***P<0.001	

* Independent t-test;

** Mann-Whitney U test;

*** Repeated Measure ANOVA;

**** Friedman

The difference in the Mean±SD total score of the development of the preterm babies in two months of adjusted age in the experimental and the control groups was statistically significant (P<0.001). Scores on the dimensions of the development scale were also compared and shown in Table 3. The Table shows that the experimental group had higher scores in all five dimensions of the development scale. 

**Table 3:** Comparison of the development scores of the preterm infants in adjusted age of two months

Dimension of scale	Mean±SD		P value
	Experimental group	Control group	
Gross motor	54.60±6.30	41.40±6.40	*P<0.001
Fine motor	56.60±4.70	41.60±8.40	*P<0.001
Communication	57.60±3.30	47.40±6.10	**P<0.001
Personal-social	58.20±3.50	53.80±5.80	**P=0.002
Problem solving	53.40±7.00	39.60±7.20	**P<0.001
Total score	280.40±15.60	223.80±22.00	**P<0.001

* Mann Whitney;

** Independent t-test

## DISCUSSION

The aim of this study was to determine the effect of home visit program based on the continuation of KMC on maternal resilience and development of premature newborns. The results of this study showed that home visit program based on the continuation of the mother and infant KMC had a positive effect on maternal resilience and newborn development. The mean resilience score of mothers one month after discharge and newborn development score at 2 months of age showed a statistically significant difference in the intervention group compared to control. In this regard, in a study, the results showed that hope therapy sessions in the intervention group increased the resilience score of the mothers. In this study, the intervention could have a positive effect on increasing the resilience score of the mothers. ^26^ Although the results of this study are consistent with those of the present study and in both studies, supportive methods were used to improve resilience, the method of increasing resilience in the present study was presented in the form of home visits. Individual visits were performed for each unit and were in accordance with mother’s specific living conditions. Mothers with premature newborn and no previous preterm birth experience were selected in this study. The continuity of the maternal-infant KMC relationship, as well as nurse-mother relationship, can affect the resilience score of the mother. In a qualitative study on the resilience of mothers with prematurely low birth weight infants, the mothers’ resilience scores were assessed by analyzing their responses to the interview. Mothers at NICU received support from the family, friends, religious counselors, and health care providers and improved their resilience. By increasing resilience, they were able to take good care of their baby and improve his/her health. The results of this study showed that the role of nurses as health care providers in the NICU in promoting mothers’ strengths and empowerment and increasing their self-confidence in caring for their infants was much more effective than other supporters. It is in line with present study in that learning the training and the mother’s ability to care for her baby at the NICU has a positive effect on the mother’s endurance, adaptability to the conditions, and her resilience. ^12^ In another study, the results showed that embracing care was associated with family resilience, while families with premature and low birth weight infants suffered from crisis and resilience disorders. ^33^ The difference is that this study qualitatively assessed the effect of family resilience on the continuation of KMC, but the present study evaluated the effect of the home visit program based on the continuation of KMC on maternal resilience and only the position of the dependent and independent variable was different in these two studies. In both studies, there has been a significant relationship between KMC and resilience, so both studies are consistent. Regarding the effect of home visit program on the maternal and infant health, resilience was compared between the intervention and control groups when the child was 5 years old. ^34^ The results showed that there were no benefits in the intervention, which contradicts the results of the present study. This discrepancy may be due to the prolongation of the intervention time; the intervening factors had an effect on the results of the study. In another study, the results showed that home visits in the intervention group had a positive effect on breastfeeding and safe infant care methods and maternal mental health. ^35^ This study is similar to the present study in terms of the study population, with the difference that in the present study, premature infants of 26-32 weeks were studied and instead of the accompanying midwife, the NICU nursing expert visited the house in two sessions until the end of the study, being in contact with research units (Mothers of premature infants) until the last follow-up on a weekly basis. Both studies confirm the effectiveness of home visits on maternal and infant outcomes.

Regarding the study of the effect of home visit program on the development of the newborn, in a study the results showed that home care program had an effect on the developmental characteristics of low birth weight infants, as the indicators of infant development in the intervention group improved. ^36^ This study is in line with the present study. However, the most change was in the dimension of problem solving and communication in their study, but the greatest difference in this study was in the dimension of fine movements and problem solving in terms of mean score. In another study, the results showed that the neonates of the KMC group rapidly reached physical growth parameters like the control neonates at the age of 40 weeks and then went beyond them and grew better while being smaller at birth. Motor growth and mental growth coefficients were also significantly better for infants in the KMC group. ^37^ Although they used a tool other than the ASQ questionnaire for developmental assessment, the results are in line with the present study and KMC intervention has improved the development of infants in the intervention group.

In Iran, the home visit system is very weak due to the need for financial and human resources and spending a lot of money to provide arrangements and preparations before and after the visit, the cost of travel, and the time spent for each client. However, according to the present study, the home visit program had a significant effect on maternal resilience and infant development and the difference in the mean scores of resilience and development in the intervention group, who received home visits, was much greater than the control group. It can be concluded that the cheapest services are not always the most effective ones, and sometimes, higfh expenditure results in much more effectiveness. For example, home visits for high-risk and vulnerable groups, such as premature infants and their mothers, can reduce the cost of treatment and the need for re-admission. Some of the limitations we encountered in this study included intrinsic characteristics of the mothers and their ability to cope with stressors and individual differences in infants.

## CONCLUSION

This study assessed the impact of home visits based on continued KMC on the development of preterm infants and resilience of their mothers. The home visits as a supportive care were successful in improving the development scores of the preterm babies and also the resilience of their mothers. Thus, inclusion of home visits after discharge in the routine care plan of preterm infants can be helpful as a national strategy. Due to the positive effects of the intervention on infant development and maternal resilience, it is recommended that the number of home visits should be increased based on the continued KMC; also to examine the infant’s development in the months after 2 months, other variables such as maternal quality of life and mother-infant attachment should be examined and measured.
